# Editorial: Nutrition in neurodegeneration: bridging diet, brain, and eye health

**DOI:** 10.3389/fnut.2026.1869273

**Published:** 2026-05-27

**Authors:** Xia Gong, Eddy Roccati, Xianwen Shang

**Affiliations:** 1 Research Centre for SHARP Vision, The Hong Kong Polytechnic University, Kowloon, Hong Kong SAR, China; 2 School of Optometry, The Hong Kong Polytechnic University, Kowloon, Hong Kong SAR, China; 3 Wicking Dementia Research and Education Centre, University of Tasmania, Hobart, TAS, Australia; 4 Collaboratory Center on Longitudinal Deep Omics, The Hong Kong Polytechnic University, Kowloon, Hong Kong SAR, China; 5 Centre for Eye and Vision Research (CEVR), Hong Kong, China

**Keywords:** age-relate macular degeneration (AMD), dementia, diet, glaucoma, neurodegeneration

Neurodegeneration represents a critical age-relate process that serves as the common pathological foundation for various brain and ocular disorders (1). Representative cerebral manifestations include Alzheimer's and Parkinson's disease, while prominent ocular types encompass glaucoma and age-related macular degeneration (AMD). Neurodegeneration affecting both the brain and the eye imposes a significant global health burden and severely impacts quality of life (2). Specifically, dementia, caused primarily by Alzheimer's disease, affected approximately 46 million people in 2015 and is estimated to increase to 131.5 million by 2050, leading to profound cognitive decline and loss of independence (3). Furthermore, the representative neurodegenerative ocular disease glaucoma exhibits devastating severity as it has resulted in 3.61 million cases of blindness and 4.14 million cases of visual impairment, accounting for 8.39% of all global blindness (4).

Growing evidence reveals numerous links between cerebral and ocular neurodegeneration as they share remarkably similar pathophysiological pathways and risk factors, which frequently lead to clinical comorbidities (2, 5, 6). The eye, as the only location in the body where neural tissue can be directly observed *in vivo*, serves as a crucial window, allowing not only the direct observation of ocular neurodegeneration but also the assessment and prediction of brain degeneration (7–10). Furthermore, brain and visual functions may interact bidirectionally, as a lack of visual stimulation could accelerate the decline in brain function (11–13).

Diet is closely linked to neurodegeneration in both the brain and the eye. For instance, a healthy diet may help stave off Alzheimer's disease (14, 15). At the same time, specific nutrients like omega-3 fatty acids and vitamin B3 have been investigated for their roles in cognitive function (16), AMD (17, 18), and glaucoma (19). Emerging research also suggests that dietary patterns, such as the Mediterranean diet, can simultaneously influence neurodegeneration in both organ systems (20).

While these findings highlight a promising intersection, the body of research on how nutrition concurrently modulates neurodegeneration in the brain and eye remains insufficient. Therefore, we launched a special focus titled “*Nutrition in neurodegeneration:* *bridging diet, brain, and eye health*” to consolidate current evidence, identify common pathways, and stimulate further investigation into this critical, interdisciplinary field. The four accepted submissions for the special focus collectively advance our understanding of nutrition's role in brain neurodegeneration, progressing from establishing robust risk factors and diagnostic tools to elucidating underlying biological pathways.

First, building on the theme of nutritional assessment in at-risk populations, the work by Alshammari et al. provides a crucial methodological validation. Their study demonstrated that the Global Leadership Initiative on Malnutrition (GLIM) criteria are a reliable and valid tool for diagnosing malnutrition in stroke survivors, showing substantial agreement with an established standard and high diagnostic accuracy (AUC, 0.819). This research addresses the foundational need for precise nutritional phenotyping, which is essential for both clinical management and research.

Regarding the association between nutrition and brain neurodegeneration, the large-scale retrospective cohort study by Huang et al. identified a clear and modifiable nutritional risk factor for dementia, reporting that zinc deficiency was associated with a 34% increased risk of new-onset dementia, with a pronounced dose-response relationship where severe deficiency elevated the risk (adjusted HR 1.71). This finding underscores the importance of specific micronutrient status in long-term brain health. Lei et al. explored the dynamic interplay between nutrition, functional mobility, and cognition in older stroke patients. Their study revealed that better nutritional status positively influenced cognitive function over 9 months. Importantly, 24.94% of this beneficial effect was indirectly mediated through improved life-space mobility. This work moves beyond association to delineate a functional pathway, showing how nutritional status affects the brain.

Delving deeper into the potential biological mechanisms underlying such diet-cognition links, Yan et al. offered insights from a population-level analysis. They found that higher dietary fiber intake was associated with better cognitive performance and more favorable inflammatory markers. Their mediation analysis further indicated that a specific inflammatory marker, the albumin-to-alkaline phosphatase ratio, accounted for 17.88% of the association between fiber intake and cognitive function. This study connects a dietary component to a plausible physiological pathway involving systemic inflammation.

While these submissions significantly advance our understanding of nutrition's role in brain neurodegeneration, they also highlight a critical gap that our special focus addresses: the concurrent investigation of the eye. Growing evidence highlights shared pathways and comorbidities between cerebral and ocular neurodegeneration, with the eye offering direct, *in vivo* observation of neural tissue. Visual function itself may modulate disease progression, as sensory deprivation can accelerate cognitive decline. We therefore call for research on how nutrition influences ocular neurodegeneration and, pivotally, identifies the common dietary and biological pathways that bridge brain and eye health. Integrating this “eye-brain” evidence is essential to advance the field from isolated interventions toward holistic, systemic neuroprotection.
